# Anti-Obesity and Anti-Diabetic Effect of Neoagarooligosaccharides on High-Fat Diet-Induced Obesity in Mice

**DOI:** 10.3390/md15040090

**Published:** 2017-03-23

**Authors:** Sun Joo Hong, Je-Hyeon Lee, Eun Joo Kim, Hea Jung Yang, Jae-Seon Park, Soon-Kwang Hong

**Affiliations:** 1 Department of Biological Science and Bioinformatics, Myongji University, 116 Myongji-Ro, Cheoin-gu, Yongin, Gyeonggido 17058, Korea; sjhong@dynebio.co.kr (S.J.H.); mscbuilding@hotmail.com (J.-S.P.); 2 Dynebio Inc., B-B205 Woolimlions Valley II, 45 Sagimagil-Ro, Jungwon-Gu, Seongnam-Si, Gyeonggi-Do 13209, Korea; jhl@dynebio.co.kr (J.-H.L.); dyne13@dynebio.co.kr (E.J.K.); hjyang@dynebio.co.kr (H.J.Y.)

**Keywords:** neoagarooligosaccharides, neoagarotetraose, neoagarohexaose, anti-obesity, antidiabetes, DagA, agar

## Abstract

Neoagarooligosaccharides (NAOs), mainly comprising neoagarotetraose and neoagarohexaose, were prepared by hydrolyzing agar with β-agarase DagA from *Streptomyces coelicolor*, and the anti-obesity and anti-diabetic effects of NAOs on high-fat diet (HFD)-induced obesity in mice were investigated after NAOs-supplementation for 64 days. Compared to the HFD group, the HFD-0.5 group that was fed with HFD + NAOs (0.5%, *w*/*w*) showed remarkable reduction of 36% for body weight gain and 37% for food efficiency ratios without abnormal clinical signs. Furthermore, fat accumulation in the liver and development of macrovesicular steatosis induced by HFD in the HFD-0.5 group were recovered nearly to the levels found in the normal diet (ND) group. NAOs intake could also effectively reduce the size (area) of adipocytes and tissue weight gain in the perirenal and epididymal adipose tissues. The increased concentrations of total cholesterol, triglyceride, and free fatty acid in serum of the HFD group were also markedly ameliorated to the levels found in serum of the ND group after NAOs-intake in a dose dependent manner. In addition, insulin resistance and glucose intolerance induced by HFD were distinctly improved, and adiponectin concentration in the blood was notably increased. All these results strongly suggest that intake of NAOs can effectively suppress obesity and obesity-related metabolic syndromes, such as hyperlipidemia, steatosis, insulin resistance, and glucose intolerance, by inducing production of adiponectin in the HFD-induced obese mice.

## 1. Introduction

Agar is the main component of the cell wall of marine red algae and is a heterogeneous polysaccharide composed of repeating units of β-1,4-d-galactopyranosyl-α-1,3-l-galactopyranose. Agar is divided into two groups—agarose and porphyran. Agarose is composed of repetitive units of agarobiose, where l-galactopyranose is replaced by 3,6-anhydro-l-galactose, with few variations and a low content of sulfate esters [1]. Porphyran is composed of repetitive units of porphyrobiose (β-1,4-d-galactose-α-1,3-l-galactose), with many variations, such as high content of 6-*O*-sulfation of the l-galactose units and 6-O-methylation of the d-galactose units [2].

**Figure 1:**
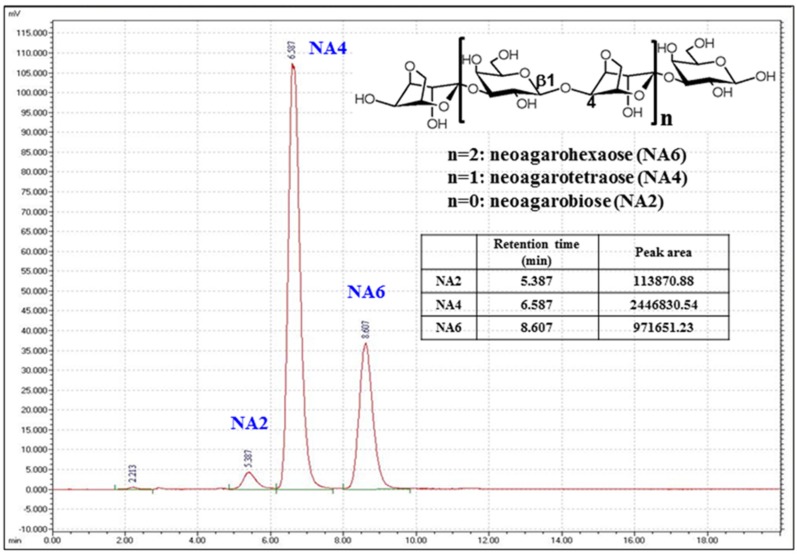
High performance liquid chromatography (HPLC) chromatograms of neoagarooligosaccharide (NAO) mixtures prepared from agar by β-agarase DagA. HPLC (Waters Corporation, Milford, MA, USA) equipped with an evaporative light scattering detector (ELSD; Sedere, Alfortville, France) was used for the analysis [6]. Column, Asahipak NH2P-50 4E multi-mode column (250 × 4.6 mm); column temperature, 40 °C; mobile phase, acetonitrile:water (65:35); flow rate, 1 mL/min; ELSD detector nebulizer temperature, 50 °C. The chemical structures of neoagarobiose (NA2), neoagarotetraose (NA4), and neoagarohexaose (NA6) are presented and each peak of individual neoagarooligosaccharide is indicated.

Possibly, agarose undergoes enzymatic hydrolysis via two pathways—α-agarolytic and β-agarolytic [3]. The α-agarolytic pathway is mediated by α-agarase (EC 3.2.1.158) that hydrolyzes the α-1,3 glycosidic bond of agarose into odd-numbered agarooligosaccharides (AOs) with 3,6-anhydro-l-galactose at their reducing end [4]. The β-agarolytic pathway is catalyzed by β-agarase (EC 3.2.1.81) that hydrolyzes β-1,4 linkages of agarose into odd-numbered neoagarooligosaccharides (NAOs) with d-galactose at their reducing end [5] (Figure 1). The degree of polymerization after enzymatic degradation is dependent on the enzyme used [3].

Agarases have wide applications in food and cosmetics industries and medical fields because they can produce oligosaccharides with remarkable biological activities [7]. In recent years, bioactivity studies have demonstrated that (N)AOs exhibit a variety of physiological activities. For instances, AOs were reported to have antitumor activity against mouse skin carcinogenesis [8], antioxidant activity, and hepatoprotective potential [9]. NAOs were reported to inhibit the growth of bacteria, slow down the degradation of starch, and be used as low-calorie additives to improve food quality [2]. NAOs also have moisturizing and whitening effects on melanoma cells [10,11]. Therefore, NAOs have enough potential for applications in food, pharmaceutical, and cosmetics industries.

Obesity is caused by an imbalance between energy intake and consumption. It is frequently associated with dyslipidemia, cardiovascular risks, hypertension, and type-2 diabetes mellitus, and thus is recognized as one of the most serious public health problems [12]. Numerous drugs targeted towards inhibition of amylase/α-glucosidase/lipase, loss of appetite, and improving fatty acid metabolism, have been approved for the treatment of obesity; however, most of them have been withdrawn from the market because of their serious adverse effects [13]. Therefore, many studies have been conducted to find and develop a new anti-obesity drug or a dietary supplement with lesser side effects [14,15].

Previously, we reported that β-agarase DagA from *Streptomyces coelicolor* was an endo-type β-agarase that degraded agarose into neoagarotetraose (NA4) and neoagarohexaose (NA6) [16]. Moreover, we optimized the heterologous production system of DagA, and then NA4 and NA6 by using the DagA to produce NAOs for in vivo experiments. In this study, we found that intake of NAOs induced anti-obesity and antidiabetic effects in high-fat diet (HFD)-induced obese mice. This is the first report on the biological efficacy of NAOs towards a metabolic syndrome.

## 2. Materials and Methods

### 2.1. Production of Streptomyces coelicolor β-Agarase DagA

The recombinant plasmid pUWL201-DagA [16] was used to overexpress the β-agarase gene (*dag*A) in *Streptomyces lividans* TK24. *S. lividans* TK24/pUWL201-DagA was maintained on R2YE agar medium [17] containing thiostrepton (25 μg/mL). RSM3 broth (glucose, 2.5%; MgCl_2_·6H_2_O, 0.5%; yeast extract, 1.1%; TES (C_6_H_15_NO_6_S, pH 7.2), 0.573%) which had been optimized for producing DagA from *S. lividans* TK24/pUWL201-DagA [6] was used for pre-cultivation (50 mL in 250-mL Erlenmeyer flask, 180 rpm, 60 h) and main fermentation (500 mL in 2-L Erlenmeyer flask, 200 rpm, 60 h) at 28 °C. The bacterial cell mass was removed by centrifugation at 10,000× *g* for 30 min at 4 °C, and the protein in the supernatant was concentrated by ammonium sulfate precipitation (85%). The precipitate was dissolved in distilled water (DW, 5 mL) and then used as the crude agarase enzyme. The agarase activity was measured by dinitrosalicylic acid (DNS) method as described previously [16]. One unit of enzyme activity was defined as the activity showing an optical density of 0.001 at 540 nm (OD_540_) after enzyme reaction in reaction buffer (20 mM Tris-HCl, pH 7.0) at 40 °C for 5 min.

### 2.2. Preparation of Neoagarooligosaccharides (NAOs)

Agar purchased from Miryang Agar Co., Ltd. (Miryang, Korea) was washed once with tap water (100 volumes of agar weight) and twice with DW (100 volumes of agar weight). The agar was dissolved in the reaction buffer (1.0%, *w/v*) by autoclaving for 15 min. After cooling down, the agar solution was kept on a shaking incubator at 43 °C, the crude DagA agarase was added (250,000 units/L) and incubated for 16 h under shaking conditions (100 rpm) at 43 °C. The reactant containing NAOs was then further purified by sequential filtration through Whatman filter paper Grade 2 (GE Healthcare, Chicago, IL, USA) and Labscale TFF (tangential flow filtration) system (5 kDa cut-off) (Millipore, Billerica, MA, USA). The final filtrate was completely lyophilized, and the NAOs powder was stored at −20 °C under dark conditions and resuspended in sterile DW before use. The fine composition of the NAOs powder was analyzed by thin layer chromatography (TLC) and high performance liquid chromatography (HPLC) as described previously [6,16].

### 2.3. Effects of NAOs Intake in High-Fat Diet (HFD)-Induced Obese Mice

This study was performed at Gyeongi Bio Center (Korea) in accordance with the guidelines established by Good Laboratory Practice (2009-183, Korea Food and Drug Administration (KFDA), 22 December 2009) and the Organization for Economic Co-operation and Development (OECD) Principles of Good Laboratory Practice (1997). The use of experimental animals was approved by Institutional Animal Care and Use Committee (IACUC) of the Gyeongi Bio Center that has been accredited by the Association for Assessment and Accreditation of Laboratory Animal Care International (AAALAC, 2010) with permission number of 2012-05-0026.

Four-week-old male C57BL/6 mice (Daehan Biolink Co. Ltd., Chungbuk, Korea) were housed individually in an air-conditioned room at 22 ± 2 °C with 50% ± 5% relative humidity and a 12 h light/dark cycle (lights on at 08:00 and lights off at 20:00), and were given a normal rodent diet (TD.94048, Purified Rodent Diet AIN-93M, Harlan Laboratories Inc., Indianapolis, IN, USA), for one week to adapt to their environment before the experiments. A HFD with 60% kcal fat primarily from lard (HFDTD.06414, Adjusted Calories Diet, Harlan Laboratories Inc.) was used to induce a rapid increase in body weight and obesity. The adapted animals were randomly divided into four groups (*n* = 8/group), where the weight difference within and between groups did not exceed ±10% of the average body weight of the sample population.

All mice were divided into normal and obese groups (*n* = 8/group) and then fed with normal diet (ND) and HFD, respectively. The HFD groups were divided into three groups according to whether they received supplemental NAOs for 64 days—the HFD group were fed HFD only, the HFD-0.25 group were fed HFD with a low dose of NAOs (0.25%, *w*/*w*), and the HFD-0.5 group were fed HFD with a high dose of NAOs (0.5%, *w*/*w*). These mice were provided with semi-synthetic diets (Table 1) and water ad libitum throughout the experimental period.

After the experimental period of 64 days, the blood was collected from the inferior vena cava after 16 h fasting. Blood samples were centrifuged and serum was separated using serum separated tube (SST) and frozen until assay. Tissue weights (liver, kidney, and epididymis) were measured and recorded as a percentage of fasted body weight and then the organs were fixed in 10% NBF (neutral buffered formalin) and frozen in liquid nitrogen.

### 2.4. Histology of Mice Adipose and Liver Tissues

The samples of mice liver and epididymis tissues were fixed using 10% NBF and embedded in paraffin. Further, 4-μm standard sections were cut and stained with hematoxylin and eosin (H&E), and were observed under an optical microscope (magnification, 100×; Olympus, Hamburg, Germany). The diagnosis of a fatty liver was made based on the presence of macro- or microvesicular fat in >5% of the hepatocytes in a H&E stained slide [18] and adipocyte size was analyzed by ImageJ program [19] developed by NIH (National Institutes of Health, Bethesda, MD, USA).

### 2.5. Biochemical Parameter Analysis

Serum triglyceride (TG) and total cholesterol (TC) were measured using an auto-chemistry analyzer (BT-2000 Plus, Biotecnica Co. Ltd., Venezia, Italy). Serum free fatty acid (FFA) was analyzed using NEFA-HR reagents (Wako Chemicals, Richmond, VA, USA) and automatic chemistry analyzer (Hitachi 7170, Hitachi High-Technologies Corporation, Tokyo, Japan). Serum insulin and adiponectin were analyzed by enzyme-linked immunosorbent assay (ELISA) kit and microplate reader (Gen5 ver2.0, BioTek Instruments Inc., Winooskii, VT, USA). Insulin-Rat/Mouse ELISA kit (EZRMI-13K, Millipore, Billerica, MA, USA) and Mouse/Rat adiponectin ELISA kit (RMADN096, SCETI, Tokyo, Japan) were used for analyses.

### 2.6. Oral Glucose Tolerance Tests (OGTT)

At 64 days, oral glucose tolerance test (OGTT) was performed as follows: 5-h fasted mice were fed 20% glucose solution by gavage (2 g glucose/kg body weight). Blood glucose concentration was determined with a glucose meter (Accu-check active, Roche, Berlin, Germany). Blood samples (3.5 μL) collected at 0, 30, 60, 90, and 120 min were used for measuring plasma glucose level [20].

### 2.7. Statistical Analysis

All numerical data were analyzed by the Student’s *t*-test to compare the data from the treatment group with those of the negative control group. The commercial statistical program, SPSS 10.1K software (IBM SPSS Statistics, San Francisco, CA, USA), was used for all statistical analyses. Significance was judged at a probability value of *p* < 0.05.

## 3. Results

### 3.1. Analysis of the Composition of NAOs Produced by β-Agarase DagA

The composition of the NAO powder, prepared by hydrolysis of agar using DagA, was analyzed by HPLC. The proportion of NA2:NA4:NA6 was 3:69:28, respectively, and the purity of NAOs in the powder was 65% (Figure 1). As the Amicon TFF ultra filtration system was used for partial purification of NAOs, the remaining portion of the powder seemed to be composed of NAOs larger than NA6, but smaller than a molecular weight (MW) of 5000 Da.

### 3.2. Effects of NAOs on Body Weight and Food Intake

**Figure 2:**
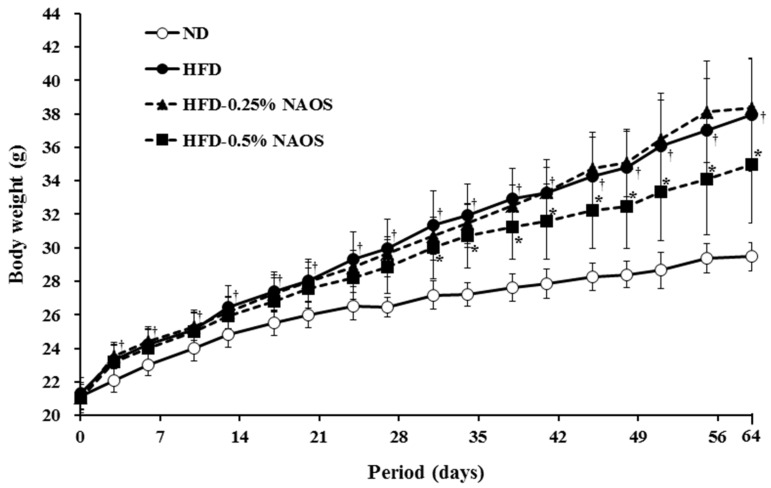
Effects of NAOs on body weight depending on treatment duration for 64 days in mice fed with high-fat diet (HFD). ND, Normal diet; HFD, High-fat diet; HFD-0.25, HFD mixed with 0.25% NAOs; HFD-0.5, HFD mixed with 0.5% NAOs. Values expressed as means ± standard error of the mean (SEM). A significant difference at *p* < 0.05 by Student’s *t*-test (*n* = 8). †, *p* < 0.05 versus normal diet; *, *p* < 0.05 versus high-fat diet.

During the 64 consecutive feeding days, mice in the four groups showed a gradual increase in body weight with different degrees (Figure 2). After 64 days, the body weight gain in the HFD group was significantly higher (16.48 g) than that in the ND group (8.22 g), indicating that HFD intake caused additional body weight gain (8.26 g) and obesity in the HFD groups. The body weight gain in the HFD-0.25 group (16.59 g) was similar to that in the HFD group (16.48 g), and that in the HFD-0.5 group (13.48 g) was significantly lower. This result indicated that supplemental intake of NAOs at a higher dose (0.5%, *w*/*w*) can effectively suppress additional body weight gain caused by HFD by 36% (Table 2).

No abnormal clinical signs were observed during the experimental period in all the groups. All the HFD groups showed low amount of food intake (65% of ND group) probably due to a high-energy density, and there was no significant difference among the HFD groups. HFD groups showed higher food efficiency ratios (FERs) than those showed by the ND group, which is a typical symptom of obesity. However, the FER was greatly reduced by NAOs intake in HFD-0.5 group, indicating that NAOs could suppress obesity induced by HFD.

### 3.3. Effects of NAOs on HFD-Induced Fatty Liver

**Figure 3:**
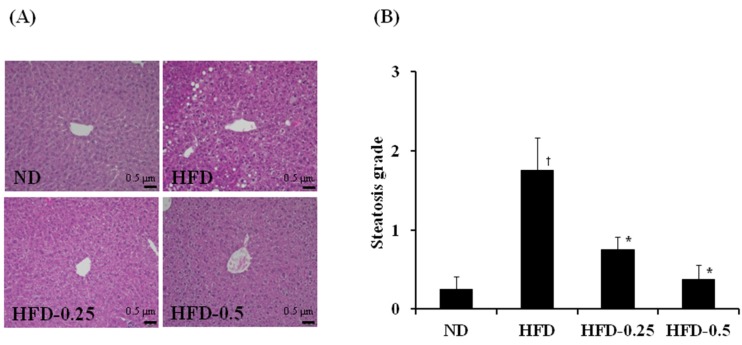
(**A**) Representative photographs showing the liver histology, and (**B**) comparison of steatosis grade [18] of the liver adipose tissue of mice fed with HFD. The tissues were surgically excised and subjected to histological analysis by staining with hematoxylin and eosin. Magnification, 100×; scale bar, 0.5 μm. ND, normal diet; HFD, high-fat diet; HFD-0.25, HFD mixed with 0.25% NAOs; HFD-0.5, HFD mixed with 0.5% NAOs. Values are means ± SEM. A significant difference at *p <* 0.05 by Student’s *t*-test (*n* = 8). †, *p* < 0.05 versus normal diet; *, *p* < 0.05 versus high-fat diet.

Although no histological abnormality was observed in the ND group, the HFD group exhibited a high degree of steatosis, showing hepatocytes with severe macrovesicular steatosis and swelling (Figure 3A). Indeed, supplementation with 0.25% and 0.5% NAOs resulted in remarkable reduction of fat deposition in hepatocytes in a dose-dependent manner. The systemic evaluation revealed that much more macrovesicular steatosis and mega-mitochondria were developed in the HFD group than in the ND group. In contrast, HFD-0.25 and HFD-0.5 groups showed reduced number and size of macrovesicular steatosis (Figure 3B). Furthermore, the HFD-0.5 group recovered the degree of fat accumulation and development of macrovesicular steatosis nearly to the state similar to that of the ND group. These histological observations of the liver tissues indicated that NAOs showed significant hepatoprotective effects in HFD-induced obese mouse model (Figure 3).

### 3.4. Effects of NAOs on Development of the Perirenal and Epididymal Adipose Tissues

**Figure 4:**
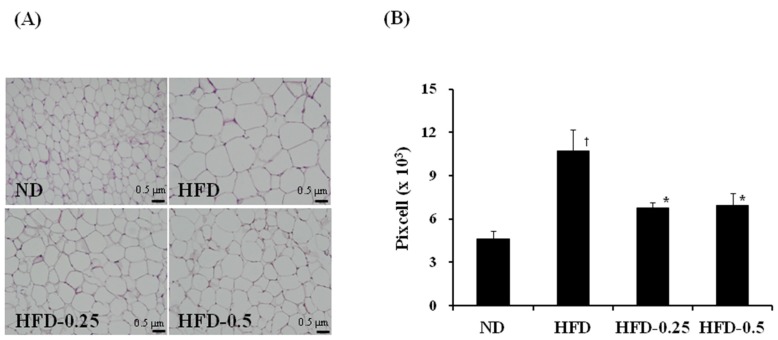
(**A**) Histological images of the epididymal adipose tissues, and (**B**) size comparison of the epididymal adipocyte from HFD-induced obese mice. The tissues were surgically excised and subjected to histological analysis by staining with hematoxylin and eosin. Magnification, 100×; scale bar, 0.5 μm. ND, normal diet; HFD, high-fat diet; HFD-0.25, HFD mixed with 0.25% NAOs; HFD-0.5, HFD mixed with 0.5% NAOs. Values are means ± SEM. A significant difference at *p <* 0.05 by Student’s *t*-test (*n* = 8). †, *p* < 0.05 versus normal diet; *, *p* < 0.05 versus high-fat diet.

The results of the histological analysis of the epididymal adipose tissue were similar to the results of the histological analysis of the liver (Figure 4), where development of the epididymal adipose tissue was greatly suppressed by NAOs intake. The area occupied by adipocytes in the perirenal and epididymal adipose tissues in the HFD group was 2.3 times higher than that of the ND group; however, the degree of increase was significantly suppressed in HFD-0.25 and HFD-0.5 groups by 66% and 64%, respectively (Table 3). The weights of the perirenal and epididymal adipose tissues from the HFD group were also noticeably increased than that of the tissues from the ND group by 4.38 and 4.06 times, respectively, which were significantly reduced in the HFD-0.5 group by 14% compared to that of HFD group. All these results strongly indicate that NAOs have an ability to suppress the development of adipose tissue induced by HFD in the liver, kidney, and epididymis, and thus prevent fat accumulation in the liver.

### 3.5. Effects of NAOs on Serum Lipid Levels

The concentrations of total cholesterol (TC), triglyceride (TG), and free fatty acid (FFA) in the serum were also remarkably higher in the HFD group compared to those in the ND group, which is also a typical symptom of obesity. However, after 64 days of treatment, the HFD-0.25 group showed significantly lower levels of serum TC, TG, and FFA. Moreover, all the three indices improved further in the HFD-0.5 group, and the TG and FFA levels were even lower than those noted in the ND group. These data clearly showed that NAOs intake can effectively mitigate hyperlipidemia induced by HFD by reducing lipid content in blood, and thus may relieve coronary artery risk factors, such as atherogenic indices (Table 4).

### 3.6. Effects of NAOs on Insulin Resistance

Because NAOs intake resulted in remarkable improvement of several indices relating to the metabolic syndrome in HFD-induced obese mice, we also tested its effects on diabetes-related indicators, such as concentrations of insulin, glucose, and adiponectin in the blood, and performed OGTT.

As shown in Table 5, concentrations of insulin and glucose in serum were significantly higher in the HFD group (188% and 133%, respectively) than that in the ND group, while concentration of adiponectin decreased to 96% of that in mice in the ND group, which indicated that HFD-induced obese mice exhibited a typical symptom of diabetes, i.e., insulin resistance. After 64 days of treatment, HFD-0.25 and HFD-0.5 groups showed significantly lower insulin level (69% of that in the HFD group). Furthermore, the concentration of adiponectin in the NAOs-supplemented groups increased in a dose dependent manner, and the HFD-0.5 group showed 122% and 127% increase compared to that in ND and HFD groups, respectively. However, the glucose concentrations in the serum were maintained at a high level in mice in all the HFD groups, probably due to high energy density (Table 5).

**Figure 5:**
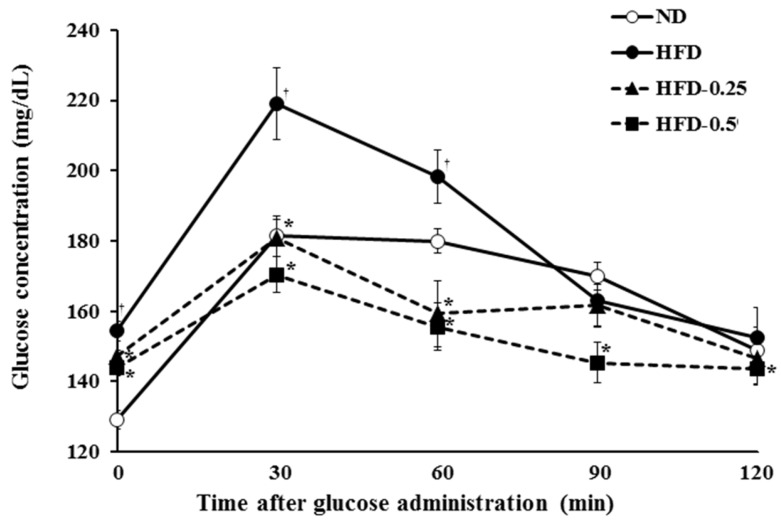
Oral glucose tolerance tests (OGTT). At 64 days, blood glucose concentrations of mice were determined at 0, 30, 60, 90, and 120 min after feeding 20% glucose solution by gavage (2 g glucose/kg body weight). ND, Normal diet; HFD, High-fat diet; HFD-0.25, HFD mixed with 0.25% NAOs; HFD-0.5, HFD mixed with 0.5% NAOs. Values are means ± SEM. A significant difference at *p <* 0.05 by Student’s *t*-test (*n* = 8). †, *p* < 0.05 versus normal diet; *, *p* < 0.05 versus high-fat diet.

The OGTT in the four groups was performed after 64 days of feeding. In the ND group, plasma glucose level reached the maximum at 30 min after glucose challenge; thereafter, a first-order kinetic of glucose elimination proceeded until 60 min (Figure 5). In contrast, there was little glucose elimination between 30 min and 60 min in the HFD group, which suggested that HFD-induced severe glucose intolerance, a symptom of diabetes. The calculated AUC (area under curve) values clearly showed that the elimination of glucose is dramatically accelerated in the HFD-0.25 and HFD-0.5 groups in a dose dependent manner, and the values are lower than those in the ND group (Table 5). All these results strongly suggest that supplementation of NAOs can improve insulin tolerance and glucose intolerance probably via increasing adiponectin concentration in the HFD-induced obese mice.

## 4. Discussion

Various oligosaccharides, such as agarooligosaccharide, chitooligosaccharide, soy oligosaccharide, and fructooligosaccharide, have been known to be biologically active with antioxidant/anti-inflammatory, hypoglycemic, and hypolipidemic effects; they allow selective growth of *Bifidobacteria* and are used as food additives [3,21,22]. In particular, agarooligosaccharides were reported to have cholesterol-lowering activity when supplemented into the diet [9].

Some medicinal plants or natural products have been reported to control glucose absorption by inhibiting the carbohydrate hydrolyzing enzymes. Thus, several α-amylase inhibitors, including acarbose, voglibose, and miglitol, are clinically used for treatment of diabetes, but they are expensive and clinical side effects often occur [23]. In the preliminary study, we investigated the effects of NAOs on α-amylase and α-glucosidase activities in vitro; however, we could not find any significant inhibitory activity of NAOs compared to that of acarbose. In addition, no inhibitory effect of NAOs on porcine pancreatic lipase was detected. Therefore, we concluded that NAOs might not act as α-amylase, α-glucosidase, or lipase inhibitors [24].

In this study, a diet-induced obesity (DIO) mouse model, where obesity was induced by feeding HFD, was used to investigate the biological function of NAOs towards metabolic syndromes, such as obesity and diabetes. Obesity is characterized by increased adipose tissue mass, due to increase in both number and size of adipocytes, and it can often cause type-2 diabetes [25,26,27,28]. Consistently, our DIO model also showed typical symptoms of obesity, such as excessive body weight increase, adipocyte increase, fatty liver, and hyperlipidemia. Moreover, it showed symptoms of pre-diabetes, such as insulin resistance, glucose intolerance, and adiponectin deficiency.

Defects in fat metabolism may cause an imbalance in energy consumption and fat combustion, which can induce pathogenesis of hepatic steatosis followed by lipid storage [29]. Xu et al. [30] reported that the liver of the DIO rodents exhibited accumulation of numerous fat droplets and the fatty liver weight was significantly higher in the HFD group than in the ND group. Consistent with the above results, our results also indicated that continuous consumption of HFD leads to hepatic steatosis associated with obesity, and supplementation of NAOs exhibited a significant hepatoprotective effect by suppressing body weight gain and ameliorating hepatic and serum lipid contents.

Adipose tissue plays a dynamic role in energy balance and consumption, and changes in mass responding to the metabolic needs of the organism [31]. The epididymal adipose tissue in mice is generally thought to be the white adipose tissue (WAT) [32], which is accumulated by excess energy intake [33]. According to our results, the weights of the epididymal and perirenal adipose tissues in NAOs-supplemented groups were significantly decreased compared to that in the HFD group. These results suggest that NAOs may prevent the accumulation of WAT in HFD-induced obese mice. However, further studies will be required to validate the relationship between anti-obesity effects of NAOs and regulation of lipogenesis-related genes in the WAT.

Adiponectin is a protein hormone that is exclusively secreted by the adipose tissue into the bloodstream and modulates a number of metabolic processes, including glucose regulation and fatty acid oxidation [34]. Serum adiponectin level is inversely related to insulin resistance, and is decreased in individuals having insulin resistance, but is increased by insulin resistance amelioration. Therefore, individuals with insulin resistance will have higher concentrations of TC, FFA, and TG in serum due to low concentration of adiponectin, leading to a fatty liver and dyslipidemia. Consistent with the above data, our data showed that serum adiponectin level was slightly lower and serum insulin level was higher in the HFD group than in the ND group, which was notably recovered in the NAOs-supplemented groups (Table 5). Moreover, the concentrations of TC, FFA, and TG, were greatly reduced to the level of the ND group by NAOs intake. The result of OGTT also strongly indicated that intake of NAOs ameliorates obesity-induced diabetes.

In conclusion, the supplementation of NAOs resulted in remarkable suppression of body weight gain, FER, and development of perirenal and epididymal adipose tissues and fatty liver, indicating that it had remarkable anti-obesity effects in DIO mice. It also greatly improved diabetes-related indices, such as dyslipidemia, insulin resistance, glucose intolerance, and adiponectin level. Taken together, we can carefully conclude that NAOs can suppress obesity and related metabolic syndromes (hyperlipidemia and pre-diabetic symptoms) by enhancing adiponectin concentration in blood, which should be further validated. In this sense, NAOs may have unique biological properties among the oligosaccharides and can be applied for preventing various metabolic syndromes in the near future.

## Figures and Tables

**Table 1:** Composition of experimental diets.

Ingredients (g/kg)	ND	HFD	HFD-0.25	HFD-0.5
Casein	210.0	230.0	230.0	230.0
l-Cystine	3.0	3.5	3.5	3.5
Maltodextrin	50.0	160.0	160.0	160.0
Sucrose	325.0	90.0	90.0	90.0
Lard	20.0	310.0	310.0	310.0
Soybean oil	20.0	30.0	30.0	30.0
Cellulose	37.15	65.5	65.5	65.5
Corn starch	280.0	-	-	-
Mineral mix	35.0	48.0	48.0	48.0
CaH(PO_4_)_2_	2.0	3.4	3.4	3.4
Vitamin mix	15.0	21.0	21.0	21.0
Choline bitartrate	2.75	3.0	3.0	3.0
Blue food color	0.1	0.1	0.1	0.1
NAOs	-	-	2.5	5
Nutrition facts: % g (*w*/*w*) (% kcal)				
Protein	18.8 (20.1)	23.5 (18.4)	23.5 (18.4)	23.5 (18.4)
Carbohydrate	64.7 (69.8)	27.3 (21.3)	27.3 (21.3)	27.3 (21.3)
Fat	4.2 (10.2)	34.3 (60.3)	34.3 (60.3)	34.3 (60.3)
Kcal/g	3.6	5.1	5.1	5.1

ND, Normal diet (TD.94048 AIN-93M Purified Diet.); HFD, High-fat diet (HFDTD.06414 Adjusted Calories Diet (60% kcal/fat)); HFD-0.25, HFD mixed with 0.25% NAOs; HFD-0.5, HFD mixed with 0.5% NAOs. Mineral mix = AIN (American Institute of Nutrition)-93G mineral mixture; Vitamin mix = AIN-93G vitamin mixture.

**Table 2:** Effects of NAOs on body weight gains, food intake, and food efficiency ratio in mice fed with high-fat diet.

Group	Body Weight (g)		Body Weight Gain (g/64 Days)	Food Intake (g/Day)	FER ^2^
	Initial	Final ^1^			
ND	21.13 ± 0.27	29.35 ± 0.27	8.22 ± 0.19	4.15 ± 0.09	2.02 ± 0.07
HFD	21.30 ± 0.30	37.78 ± 1.04 ^a^	16.48 ± 0.85 ^a^	2.74 ± 0.05 ^a^	6.40 ± 0.51 ^a^
HFD-0.25	21.00 ± 0.25	37.59 ± 0.63	16.59 ± 0.58	2.56 ± 0.10	6.45 ± 0.36
HFD-0.5	20.89 ± 0.27	34.37 ± 0.97 ^b^	13.48 ± 0.94 ^b^	2.77 ± 0.01	4.82 ± 0.36 ^b^

ND, Normal diet; HFD, High-fat diet; HFD-0.25, HFD mixed with 0.25% NAOs; HFD-0.5, HFD mixed with 0.5% NAOs. ^1^ The data were obtained before carrying out oral glucose tolerance tests at 64 days of experiments. ^2^ FER (food efficiency ratio), body weight gain (g)/food intake (g). Values expressed as means ± SEM. A significant difference at *p* < 0.05 by Student’s *t*-test (*n* = 8). ^a^ A significant decrease at *p* < 0.05 versus normal diet. ^b^ A significant decrease at *p* < 0.05 versus high-fat diet.

**Table 3:** Effects of NAOs on development of perirenal and epididymal adipose tissues in mice fed with high-fat diet.

Group	Adipocyte Size (Pixel × 10^3^)	Perirenal Adipose Tissue (g)	Epididymal Adipose Tissue (g)
ND	4.64 ± 0.54	0.215 ± 0.035	0.222 ± 0.031
HFD	10.74 ± 1.44 ^a^	0.920 ± 0.329 ^a^	0.902 ± 0.312 ^a^
HFD-0.25	6.74 ± 0.38 ^b^	0.929 ± 0.149	0.923 ± 0.201
HFD-0.5	6.95 ± 0.82 ^b^	0.824 ± 0.413	0.797 ± 0.370

ND: Normal diet; HFD: High-fat diet; HFD-0.25: HFD mixed with 0.25% NAOs; HFD-0.5: HFD mixed with 0.5% NAOs. Values are means ± SEM. A significant difference at *p <* 0.05 by Student’s *t*-test (*n* = 8). ^a^ A significant decrease at *p* < 0.05 versus normal diet. ^b^ A significant decrease at *p* < 0.05 versus high-fat diet.

**Table 4:** Effects of NAOs on serum lipid concentration in mice fed with high-fat diet.

Group	Total Cholesterol (mg/dL)	Triglyceride (mg/dL)	Free Fatty Acid (mEq/L ^1^)
ND	140.1 ± 5.2	48.7 ± 3.2	1.28 ± 0.07
HFD	182.0 ± 8.2 ^a^	64.8 ± 4.0 ^a^	1.52 ± 0.11 ^a^
HFD-0.25	173.0 ± 4.3	56.7 ± 4.4	1.30 ± 0.05 ^b^
HFD-0.5	159.6 ± 5.6 ^b^	47.3 ± 2.9 ^b^	1.22 ± 0.06 ^b^

ND, Normal diet; HFD, High-fat diet; HFD-0.25, HFD mixed with 0.25% NAOs; HFD-0.5, HFD mixed with 0.5% NAOs. **^1^** Milliequivalents per liter. Values are means ± SEM. A significant difference at *p* < 0.05 by Student’s *t*-test (*n* = 8). ^a^ A significant decrease at *p* < 0.05 versus normal diet. ^b^ A significant decrease at *p* < 0.05 versus high-fat diet.

**Table 5:** Effect of NAOs on concentrations of insulin, glucose, and adiponectin in serum of mice fed with high-fat diet.

Group	Area under Curve (mg, 0–120 min)	Insulin (ng/mL)	Glucose (mg/dL)	Adiponectin (ng/mL)
ND	332.3 ± 6.8	0.72 ± 0.12	115.4 ± 7.3	5.01 ± 0.79
HFD	373.1 ± 12.4 ^a^	1.35 ± 0.14 ^a^	153.3 ± 7.3 ^a^	4.81 ± 1.11
HFD-0.25	322.4 ± 11.6 ^b^	0.92 ± 0.13 ^b^	159.4 ± 4.0	5.63 ± 0.81
HFD-0.5	308.0 ± 10.6 ^b^	0.94 ± 0.14	156.8 ± 19.6	6.13 ± 1.25 ^b^

ND, Normal diet; HFD, High-fat diet; HFD-0.25, HFD mixed with 0.25% NAOs; HFD-0.5, HFD mixed with 0.5% NAOs. Values are means ± SE. A significant difference at *p <* 0.05 by Student’s *t*-test (*n* = 8). ^a^ A significant decrease at *p* < 0.05 versus normal diet. ^b^ A significant decrease at *p* < 0.05 versus high-fat diet.
